# Numerical analysis of turbulent flow characteristics with the influence of speed ratio in a double-sided cavity

**DOI:** 10.1016/j.mex.2024.102594

**Published:** 2024-02-01

**Authors:** Manogaran Gnanasekaran, Anbalagan Satheesh

**Affiliations:** Department of Thermal and Energy Engineering, School of Mechanical Engineering, Vellore Institute of Technology, Vellore, Tamilnadu 632014, India

**Keywords:** Speed ratio, k-ε turbulence model, Turbulent viscosity, k-ε turbulence models with finite volume method

## Abstract

The present study numerically investigates the two-dimensional steady incompressible turbulent flow characteristics in an enclosed cavity. The finite volume method (FVM) is used to discretize the governing equations, and k-ε turbulence models are adopted to predict the flow characteristics. The turbulent flow behavior is studied by varying the speed ratio (0.05 ≤ *S* ≤ 1.0), aspect ratio (0.5 ≤ *K* ≤ 2.0), and Reynolds number (1 × 10^4^ ≤ *Re* ≤ 2 × 10^5^). The flow characteristics are analyzed using stream function (ψ), Reynolds stresses (u'v'), and turbulent quantities. Results show the Reynolds number and speed ratio significantly influence the formation of vortices over the selected range of operating parameters. With the speed ratio, the turbulent kinetic energy reduces considerably by increasing the Reynolds number and aspect ratio. Similarly, for *S* = 0.05 and *K* = 0.5, the turbulent kinetic energy and dissipation rate are decreased by 89.16% and 42.28%, respectively. When *Re* is increased from 1 × 10^4^ to 2 × 10^5^, the turbulent viscosity increases by 92.10%. By comparing the results, average turbulent quantities are decreased by increasing the flow parameters.

• Turbulent flow behavior is investigated by using the FVM near-wall treatment approach.One of the unique parameters called speed ratio is emphasized.
• Contours of turbulence kinetic energy, dissipation, and viscosity are examined.
• The average intensity of turbulent quantities is decreased by increasing the speed ratio.

## Graphical abstract

**Figure:**
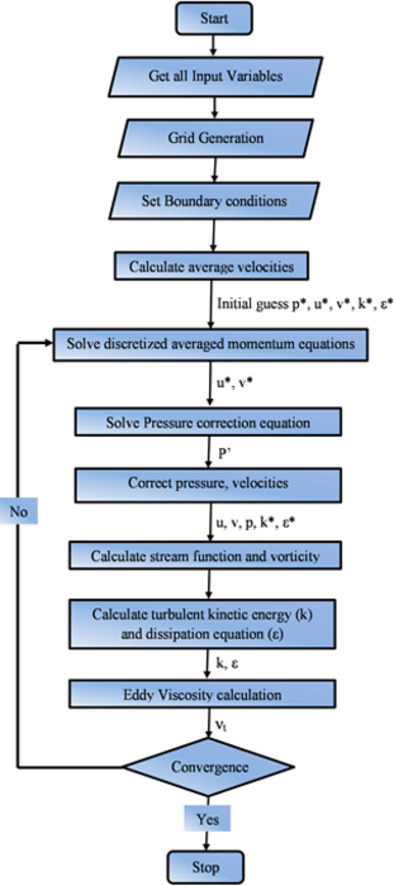

## Nomenclature

E_1_: linear coefficient
G: production term
*H*: reference length(m)
TKE: turbulent kinetic energy
K: aspect ratio
P: pressure (N/m^2^)
$\bar{p}$: mean pressure components
P: non- dimensional pressure components
*_Re_*: Reynolds number
S: speed ratio
u_τ_: frictional velocity (m/s)
u,v: velocity components (m/s)
U, V: non- dimensional velocity components
$\bar{u},\bar{v}$: mean velocity components
$u^{\prime },v^{\prime }$: fluctuating velocity components
$\bar{u_{i}^{\prime }u_{j}^{\prime }}$: Reynolds average stress
x,y: Cartesian coordinates (m)
X, Y: non- dimensional coordinates
*y*^+^: wall function

**Greek symbols**
ε: dissipation rate
ρ: density (kg/m^3^)
μ: dynamic viscosity (N/m^2^s)
ϑ: kinematic viscosity (m^2^/s)

**Subscripts**
*B*: bottom
*i,j*: vector direction
*N*: non-dimensional
*t*: turbulent
*T*: top

Specifications table

**Table:** 

Subject area:	Engineering
More specific subject area:	Mechanical Engineering, Computational Fluid Dynamics
Name of your method:	k-ε turbulence models with finite volume method
Name and reference of original method:	New contribution
Resource availability:	Made on request

**Method details**

### Introduction

A lid-driven cavity was observed as one of the significant problems in examining the flow stability. Due to their ability to be done for an extensive range of Reynolds numbers, the cavity problems received special attention. Numerous experimental and computational studies have been carried out by varying the speed ratio, Reynolds number, aspect ratio, mathematical formulations, wall movement, and various numerical approaches in cavity flow. Double-sided cavities have evolved in several engineering applications to meet industrial and technological needs. They are fluid flow, including solar thermal systems, building cooling and heating, heat exchangers, room ventilation, thermal energy storage, cooling of electronic devices, fuel cells, geothermal systems, storage tanks, coating systems, mixing due to chaotic advection, and drying methods make this field of study fascinating [1], [2], [3], [4]. In addition, Hammami et al. [5] provided various lid-driven flow cavity applications that have always attracted much interest, including electronic card cooling, food processing, multi-screen nuclear reactor structures, and crystal formation. Meniscus roll-coating and polymer processing both involve this cavity problem when horizontal rollers move at the same or different rates [6]. The double-sided lid-driven cavity model with various speed ratios in turbulent flow has several potential impacts and industrial applications, particularly in drainage management. The benefits of this technology in terms of drainage management lie in its adaptability to different conditions. By dynamically adjusting speed ratios, it can optimize fluid removal, prevent stagnation, and reduce the risk of blockages in drainage systems. Overall, the double-sided cavity model offers a versatile and efficient approach to fluid dynamics that can be tailored to various real-world applications, contributing to better drainage and environmental sustainability***.***As mentioned earlier, the physical processes involved in the applications can be understood and investigated effectively using numerical techniques. It demonstrates that numerous researchers are under investigation utilizing a variety of experiments. However, conducting exhaustive experiments for all the analyses might not be practical. Numerical studies on lid-driven cavities have been carried out to validate the experimental results or better understand the cavity flows. Numerical studies have significantly developed in recent years due to the advancement in computational resources.

Shankar et al. [7] examined the significance of cavities sophisticatedly. With the movement of the wall in basic shapes, they demonstrated practically all internal recirculating fluid flow processes. Longitudinal vortices, corner eddies, non-uniqueness, transition, and turbulence may be investigated in the same confined geometries since they occur naturally. Ghia et al. [8] and Erturk et al. [9] have the most often cited publications in the square cavity for laminar flow for *Re* = 1 × 10^4^ and *Re* = 2 × 10^4^, respectively. Significant numerical investigations have also been on single lid-driven cavities with uniform wall movement [10], [11], [12], [13], [14], [15], [16]. The studies mentioned above solely investigated the single-sided cavity. However, many researchers have examined two-sided cavity problems. Kuhlmann et al. [17] extended the research to double-sided cavities using numerical and experimental techniques to study 2D and 3D flows in cavities with walls moving in the opposite direction. The results demonstrated that the cavity aspect ratio and side wall velocities significantly impact the vortex shape. The following section discusses some significant numerical research on double-sided cavities.

At a Reynolds number of 1200, Blohm et al. [18] explored steady and unsteady flows in the motion of two opposing sides in a rectangular chamber. Gaskell et al. [19] studied stokes flow for various speed ratios (−1.0 to 1.0) and aspect ratios (0.5 to 2.0) in a double-sided cavity. A wide variety of flow configurations are demonstrated for both modes of speed ratio operation. Wahba et al. [20] numerically investigated the double and four-sided cavities in a 2D incompressible flow. For *Re* = 10, the double and four-sided driven cavity diagonals are symmetric about the flow field. Chen et al. [21] examined the bifurcation flow in a double-sided cavity with walls that moved in the reverse direction for Reynolds numbers (1≤ *Re* ≤ 1200) and aspect ratios (1.0 ≤ *K* ≤ 2.5). However, they are all examined by resolving the equation with the laminar flow. Hammami et al. [22] explored the bifurcation principle with the effects of aspect and speed ratios in a two-sided cavity. The speed and aspect ratio ranges were 0.25 to 0.825 and 0.25 to 1.0, respectively. Souayeh et al. [23] examine the impact of the semicircle having a radius (0.1 to 0.25) positioned at the bottom side of the cavity with varying speed ratio (0 to 1) at *Re* = 1000. Substantial changes in the flow features have been observed when the radius is varied. Mendu et al. [24] investigated the power law with non-Newtonian fluid in a double-side cavity. They examined the impacts of the Reynolds number, speed ratio, and power-law index. The results showed that the power-law index varies linearly with the drag coefficient, and the intensity of the secondary vortices is reduced. CheSidik et al. [25] evaluated the double-sided lid-driven cavity in 2D, incompressible flow configuration at various speed ratios (0 - 1.0) and Reynolds numbers (100 - 1000). For the effects of speed ratio and Reynolds number, they found changes in the location of the primary vortex. The literature above demonstrates the significance of aspect and speed ratios in cavity problems. However, as mentioned earlier, the investigations discuss laminar flow characteristics. The following research studies cover cavity flow problems using various turbulence modeling techniques.

Numerous lid-driven cavity investigations reported in the literature treat the flow when *Re* = 1 × 10^4^ as laminar [8,^9^,[26], [27], [28], [29], [30]. Several analyses use different turbulence models, starting with the Reynolds number from 3200 [31], [32], [33], [34], [35]. Hence, the flow characteristics and the performance have to be thoroughly investigated. For a 2D lid-driven cavity problem, Nagapetyan et al. [36] explored solving the various turbulence modeling equations. According to their conclusion, the newly designed Wray-Agarwal model performs effectively for the chosen Reynolds numbers compared to the existing turbulence models. With different span-wise and depth-wise aspect ratios, Samantaray et al. [37] investigated the multiple aspect ratios of turbulent flow with high Reynolds numbers inside the cavity. As the aspect ratio increases, the secondary vortex gets bigger. Patel et al. [38] numerically studied the incompressible turbulent flow with horizontal cavity walls moving in the anti-parallel motion for *Re*=12,000. They provided the time history and the power spectrum for variables, including turbulent kinetic energy and turbulent production, for the zone with the highest turbulence creation. The turbulent flow parameters for *Re*=2 × 10^4^ to *Re*=5 × 10^4^ in a passive rectangular cavity were recently studied by Kumar et al. [39]. They could identify the flow structure by analyzing the shear layer growth rate, velocity gradients, velocity profiles, and turbulence features.

Furthermore, many papers investigated the turbulent flows in the double-sided cavity using advanced simulation techniques. Direct Numerical Simulation (DNS) was used by Leriche et al. [40] to simulate the turbulent flow conditions for Reynolds numbers more significant than 1 × 10^4^. The turbulence in a 3D shear-driven cavity was numerically examined by Jordan et al. [32]. For *Re* = 5000 and *Re* = 10,000, they employed DNS and Large Eddy Simulation (LES), respectively. They also identified new flow behavior inside the cavity. Another study by Jordan et al. [41] observed the maximum level of turbulence formation using Large Eddy Simulations and dynamic modeling. Bouffanais et al. [42] examined the turbulent flow using LES and the spectral element approach in a lid-driven cavity at *Re*=12,000.They studied and evaluated two sub-grid models that also showed the characteristics of the turbulent regime.

Various numerical models, such as RANS, LES, DNS, etc., are used to solve turbulent nonlinear equations. The most accurate method for modeling turbulent flow uses DNS to solve the Navier-Stokes equations and seek a comprehensive three-dimensional resolution of all turbulent scales in time and space. Nevertheless, DNS is costly and limited to low Reynolds number flows over basic geometries. Large eddies in turbulent flow were the only movements that LES could resolve. Even though the model is less costly than DNS, most practical applications require too much processing effort and resources. The RANS model is an alternate method for simulating turbulent flow, and RANS has models for all turbulent length scales. Due to its less expensive computational efficiency, industrial applicability, robustness, boundary layer prediction, simplicity and accessibility, it has been the foundation of the contemporary CFD approach for modeling turbulent flow over the past few decades [43], [44], [45].

More researchers have researched this topic for laminar flow at Reynolds numbers ranging from 100 to 1 × 10^4^, depending on the Reynolds numbers, speed ratio, and cavity dimensions [8,^9^,[23], [24], [25],46]. Only a few numerical studies on the turbulent flow at Reynolds number greater than 1 × 10^4^ are reported [31], [32], [33], [34], [35] and the effect of speed ratio in the turbulent flow is not much studied.According to the author, no one has investigated the turbulent flow behavior in the cavity with speed ratio. Therefore, this study examines the turbulent flow characteristics in the 2D double-sided cavity using the RANS equation with the k-ε turbulence model.

### Mathematical modeling

**Fig. 1:**
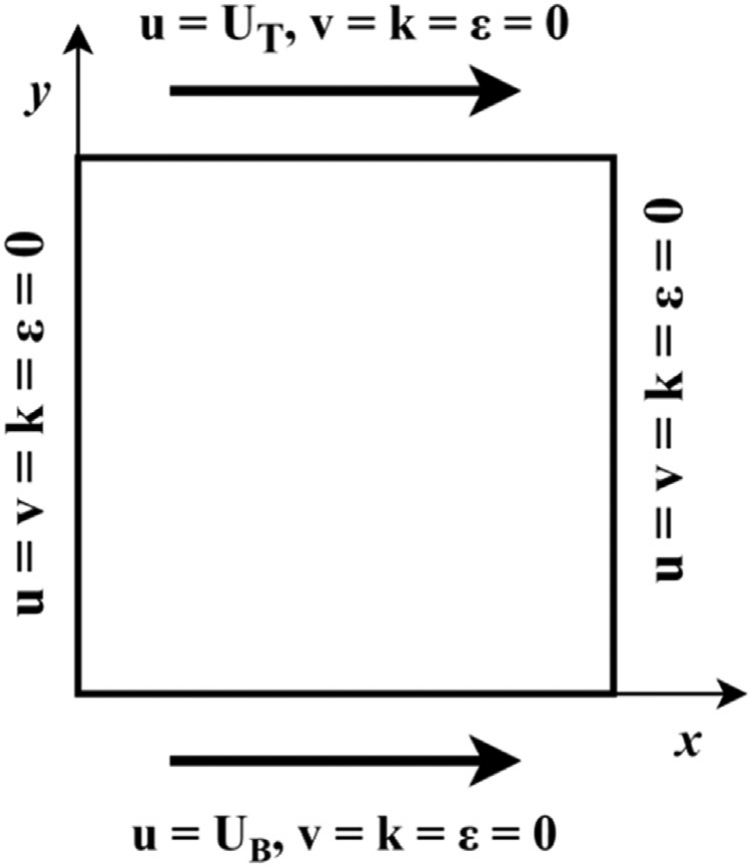
Mathematical model.

The mathematical model used in the current study is shown in Fig. 1. The boundary conditions are maintained with the horizontal walls moving toward the positive X direction, and the side walls are constant (*u* = *v* = 0). In contrast, the top and bottom walls move with a velocity of U_T_ and U_B_, respectively. By varying the speed ratio (0.05 - 1.0), aspect ratio (0.5 - 2.0), and Reynolds Number (1 × 10^4^–2 × 10^5^), the flow patterns were observed and analyzed.

#### Governing equations

The flow of 2D steady-state and incompressible turbulent mean flow characteristics are predicted using the following set of RANS governing equations with the two-equation k-ε turbulence model. A thorough examination of the turbulence modeling performed in the current investigation is provided by Biswas et al. [47]. The Boussinesq approximation relates the Reynolds stresses to the velocity gradients. The mean pressure and velocity components represent $\bar{p}$ and $\bar{u},\bar{v}$ respectively. The governing equations for 2D incompressible turbulent flow are given in Eqs. (1) to (3).

(1) $$\frac{\partial \bar{u}}{\partial x}+\frac{\partial \bar{v}}{\partial y}=0$$

(2) $$\frac{\partial \bar{u}\bar{u}}{\partial x}+\frac{\partial \bar{u}\bar{v}}{\partial y}=-\frac{1}{\rho }\frac{\partial \bar{p}}{\partial x}+\frac{\partial }{\partial x}\left[\vartheta \frac{\partial \bar{u}}{\partial x}-\bar{u^{\prime }u^{\prime }}\right]+\frac{\partial }{\partial y}\left[\vartheta \frac{\partial \bar{u}}{\partial y}-\bar{u^{\prime }v^{\prime }}\right]$$

(3) $$\frac{\partial \bar{u}\bar{v}}{\partial x}+\frac{\partial \bar{v}\bar{v}}{\partial y}=-\frac{1}{\rho }\frac{\partial \bar{p}}{\partial y}+\frac{\partial }{\partial x}\left[\vartheta \frac{\partial \bar{v}}{\partial x}-\bar{u^{\prime }v^{\prime }}\right]+\frac{\partial }{\partial y}\left[\vartheta \frac{\partial \bar{v}}{\partial y}-\bar{v^{\prime }v^{\prime }}\right]$$

Where the average velocities and pressure are denoted by $\bar{u}$,$\bar{v}$, and $\bar{p}$ respectively. Similarly, kinematic viscosity is ν, and the fluctuating velocities are $u^{\prime }$,$v^{\prime }$. Using eddy viscosity to calculate Reynolds stresses$\left(-\bar{u_{i}^{\prime }u_{j}^{\prime }}\right)$, Boussinesq developed an approximation for the turbulence stresses to mean flow [47].

(4) $$-\bar{u_{i}^{\prime }u_{j}^{\prime }}=\vartheta_{t}\left[\frac{\partial \bar{u_{i}}}{\partial x_{j}}+\frac{\partial \bar{u_{j}}}{\partial x_{i}}\right]-\frac{2}{3}k\delta_{ij}$$

δ_ij_ and k are the Kronecker delta and turbulent kinetic energy, respectively. Substitute Eq. (4) in Eqs. (2) and (3), which becomes

(5) $$\frac{\partial \bar{u}\bar{u}}{\partial x}+\frac{\partial \bar{u}\bar{v}}{\partial y}=-\frac{1}{\rho }\frac{\partial \bar{p}}{\partial x}+\frac{\partial }{\partial x}\left[\left(\vartheta +\vartheta_{t}\right)\frac{\partial \bar{u}}{\partial x}-\frac{2}{3}k\right]+\frac{\partial }{\partial y}\left[\left(\vartheta +\vartheta_{t}\right)\frac{\partial \bar{u}}{\partial y}\right]$$

(6) $$\frac{\partial \bar{u}\bar{v}}{\partial x}+\frac{\partial \bar{v}\bar{v}}{\partial y}=-\frac{1}{\rho }\frac{\partial \bar{p}}{\partial y}+\frac{\partial }{\partial x}\left[\left(\vartheta +\vartheta_{t}\right)\frac{\partial \bar{v}}{\partial x}\right]+\frac{\partial }{\partial y}\left[\left(\vartheta +\vartheta_{t}\right)\frac{\partial \bar{v}}{\partial y}-\frac{2}{3}k\right]$$

The k-ε turbulence model is proposed by Launder et al. [48]. It is the most popular model used to calculate the mean flow properties of turbulence. In that turbulent kinetic energy (k) equation can be written as

(7) $$\frac{\partial \bar{u}k}{\partial x}+\frac{\partial \bar{v}k}{\partial y}=\frac{\partial }{\partial x}\left[\left(\vartheta +\frac{\vartheta_{t}}{\sigma_{k}}\right)\frac{\partial k}{\partial x}\right]+\frac{\partial }{\partial y}\left[\left(\vartheta +\frac{\vartheta_{t}}{\sigma_{k}}\right)\frac{\partial k}{\partial y}\right]+G-\varepsilon$$

The terms diffusion, production, and dissipation in kinetic energy are on the right side of the equation above, with the advection term on the left. The rate of dissipation (ε) equation can be written as

(8) $$\frac{\partial \bar{u}\varepsilon }{\partial x}+\frac{\partial \bar{v}\varepsilon }{\partial y}=\frac{\partial }{\partial x}\left[\left(\vartheta +\frac{\vartheta_{t}}{\sigma_{\varepsilon }}\right)\frac{\partial \varepsilon }{\partial x}\right]+\frac{\partial }{\partial y}\left[\left(\vartheta +\frac{\vartheta_{t}}{\sigma_{\varepsilon }}\right)\frac{\partial \varepsilon }{\partial y}\right]+C_{1\varepsilon }\frac{\varepsilon }{k}G-C_{2\varepsilon }\frac{\varepsilon^{2}}{k}$$

Where G and $\vartheta_{t}$ are represented as

$$\vartheta_{t}=C_{\mu }\frac{k^{2}}{\varepsilon }$$

$$G=\vartheta_{t}\left[2\left[{\left(\frac{\partial \bar{u}}{\partial x}\right)}^{2}+{\left(\frac{\partial \bar{v}}{\partial y}\right)}^{2}\right]+{\left(\frac{\partial \bar{u}}{\partial y}+\frac{\partial \bar{v}}{\partial x}\right)}^{2}\right]$$

G, $\vartheta_{t}$, ϑ, and ρ denote production by shear, turbulent viscosity, kinematic viscosity, and density, respectively. The non-dimensional form is expressed as follows:

$$\bar{U}=\frac{\bar{u}}{U_{0}};\;\bar{V}=\frac{\bar{v}}{U_{0}};\;X=\frac{\bar{x}}{h};\;Y=\frac{\bar{y}}{h};\;\bar{P}=\frac{\bar{p}-\bar{p_{0}}}{\rho U_{0}^{2}};\;k_{n}=\frac{k}{U_{0}^{2}};\;\varepsilon_{n}=\frac{\varepsilon }{U_{0}^{3}/h};\;\vartheta_{t,n}=\frac{\vartheta_{t}}{\vartheta };\;Re=\frac{U_{0}l}{\vartheta }$$

Substituting the above non-dimensional terms into Eqs. (1) and (5) to (8), the obtained non-dimensional equation is written as

(9) $$\frac{\partial \bar{U}}{\partial X}+\frac{\partial \bar{V}}{\partial Y}=0$$

(10) ∂U‾U‾∂X+∂U‾V‾∂Y=−∂∂X[P‾+23k]+1Re∂∂X[(1+ϑt,n)∂U‾∂X]+1Re∂∂Y[(1+ϑt,n)∂U‾∂Y]

(11) ∂U‾V‾∂X+∂V‾V‾∂Y=−∂∂Y[P‾+23k]+1Re∂∂X[(1+ϑt,n)∂V‾∂X]+1Re∂∂Y[(1+ϑt,n)∂V‾∂Y]

(12) $$\frac{\partial \bar{U}k_{n}}{\partial X}+\frac{\partial \bar{V}k_{n}}{\partial Y}=\frac{1}{Re}\frac{\partial }{\partial X}\left[\left(1+\frac{\vartheta_{t,n}}{\sigma_{k}}\right)\frac{\partial k_{n}}{\partial X}\right]+\frac{1}{Re}\frac{\partial }{\partial Y}\left[\left(1+\frac{\vartheta_{t,n}}{\sigma_{k}}\right)\frac{\partial k_{n}}{\partial Y}\right]+G_{n}-\varepsilon_{n}$$

(13) $$\frac{\partial \bar{U}\varepsilon_{n}}{\partial X}+\frac{\partial \bar{V}\varepsilon_{n}}{\partial Y}=\frac{1}{Re}\frac{\partial }{\partial X}\left[\left(1+\frac{\vartheta_{t,n}}{\sigma_{\varepsilon }}\right)\frac{\partial \varepsilon_{n}}{\partial X}\right]+\frac{1}{Re}\frac{\partial }{\partial Y}\left[\left(1+\frac{\vartheta_{t,n}}{\sigma_{\varepsilon }}\right)\frac{\partial \varepsilon_{n}}{\partial Y}\right]+C_{1\varepsilon }\frac{\varepsilon_{n}}{k_{n}}G_{n}-C_{2\varepsilon }\frac{\varepsilon_{n}^{2}}{k_{n}}$$

(14) $$G=\frac{\vartheta_{t,n}}{Re}\left[2\left[{\left(\frac{\partial \bar{U}}{\partial X}\right)}^{2}+{\left(\frac{\partial \bar{V}}{\partial Y}\right)}^{2}\right]+{\left(\frac{\partial \bar{U}}{\partial Y}+\frac{\partial \bar{V}}{\partial X}\right)}^{2}\right]$$

(15) $$\vartheta_{t,n}=C_{\mu }Re\frac{k_{n}^{2}}{\varepsilon_{n}}$$

The k-ε model constants used in the above equations from Biswas et al. [47] can be given by:

$$\sigma_{k}=1.0,\sigma_{\varepsilon }=1.30,C_{1\varepsilon }=1.44,C_{2\varepsilon }=1.92,C_{\mu }=0.09$$

With the help of the wall function (*y*^+^), the mathematical problem may be defined with boundary conditions. Even though fine mesh simulation provides the most accurate results, the computation time required is exceptionally high. So, a wall function that connects the turbulent zone with the entire wall layer is used. The initial computational point (p) is close to the wall in the fully turbulent log-law zone. The following relationships determine the friction velocity (U_τ_) [49].

(16) $$y_{p}^{+}=\frac{y_{p}U_{\tau }}{\vartheta };\;\frac{U_{p}}{U_{\tau }}=\frac{1}{k}\ln\left(Ey_{p}^{+}\right);\;k_{p}=\frac{U_{\tau }^{2}}{\sqrt{C_{\mu }}};\;\varepsilon_{p}=\frac{U_{\tau }^{3}}{ky_{p}}$$

where, Von Karman constant (*k* = 0.41) and linear coefficient (*E* = 9.0). Similarly, U_p_, k_p,_ and ε_p_ represent wall velocity, kinetic energy, and dissipation rate at the point y_p_.

#### Boundary conditions

The boundary conditions are maintained with horizontal walls moving in the positive X direction, with vertical walls stationary, as shown in Table 1.

**Table 1:** Present study Boundary conditions.

Wall	Boundary Conditions	
Bottom	$u=U_{B};v=0;k=0;\varepsilon =0$	y=0;0≤x≤L
Top	$u=U_{T};v=0;k=0;\varepsilon =0$	y=H;0≤x≤L
Left	u=0;v=0;k=0;ε=0	x=0;0≤y≤H
Right	u=0;v=0;k=0;ε=0	x=L;0≤y≤H

### Numerical study

#### Solution technique

**Fig. 2:**
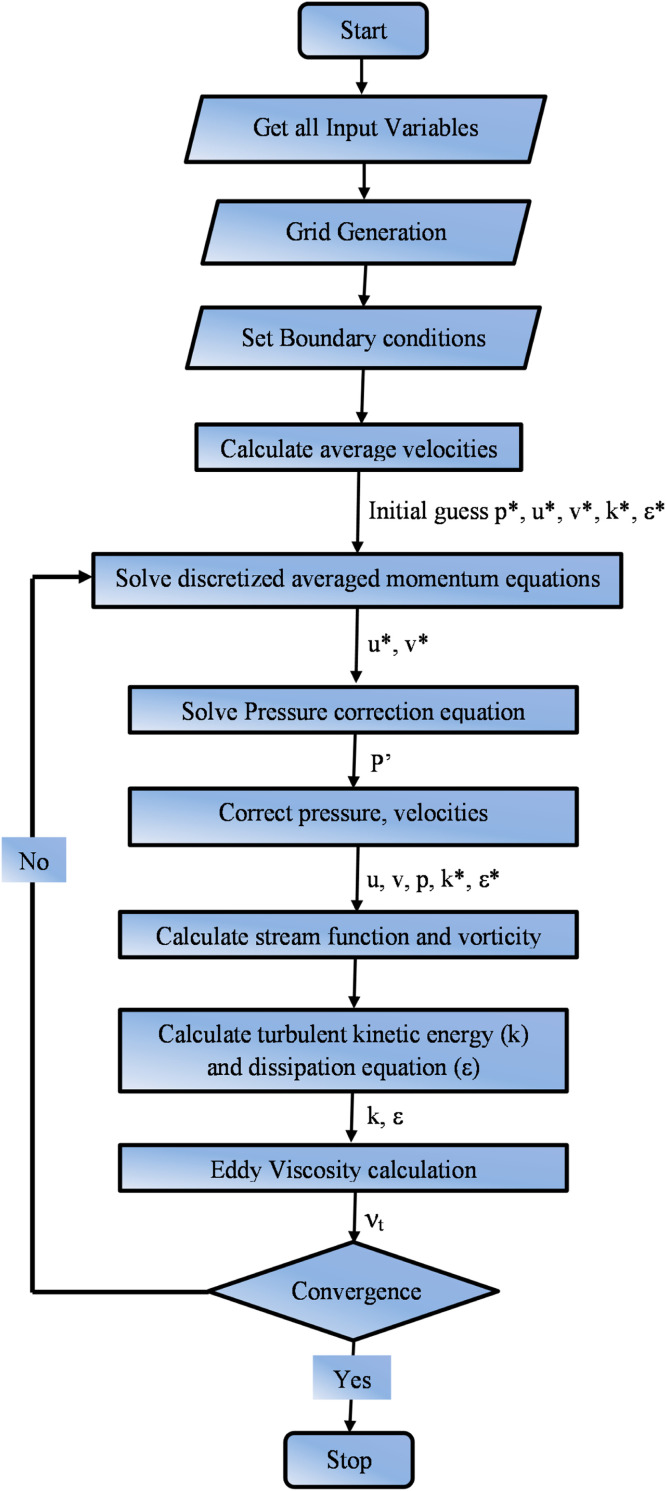
Flow diagram.

The commercial solver C++ code performs computation using the current mathematical model. The governing equations are solved by a staggered grid system using FVM [50]. The present simulation flow diagram is shown in Fig. 2. A fine rectangular mesh with a uniform grid size discretizes the entire domain while considering the wall effect. The momentum and continuity equations are linked using the SIMPLE algorithm. The diffusion and convection terms are discretized using Hybrid and Quadratic Upstream Interpolation for Convective Kinematics (QUICK) Schemes [51]. The Momentum and pressure correction equations are solved using the TDMA and Gauss Siedel solver. Solving nonlinear equations involves transforming the discretized equations into linear form, solving the resultant linear equations, and obtaining updated values for the dependent variables.

A numerical study is currently being conducted to investigate how aspect ratio, speed ratio, and Reynolds number affect the flow characteristics of a 2D steady-state turbulent flow within a cavity. The parameter ranges are shown in Table 2. The iterations are continued until the following criterion is met to ensure convergence of the numerical model.

$$\frac{\sum_{i,j}\left|x_{i,j}^{new}-x_{i,j}^{old}\right|}{\sum_{i,j}\left|x_{i,j}^{new}\right|}\leq 10^{-8}$$

**Table 2:** Range of Parameters.

Parameters	Range				
Aspect Ratio (K)	0.5	1.0	2.0	–	–
Speed Ratio (S)	0.05	0.25	0.5	1.0	–
Reynolds Number (*Re*)	1 × 10^4^	5 × 10^4^	1 × 10^5^	1.5 × 10^5^	2 × 10^5^

#### Grid convergence study

**Fig. 3:**
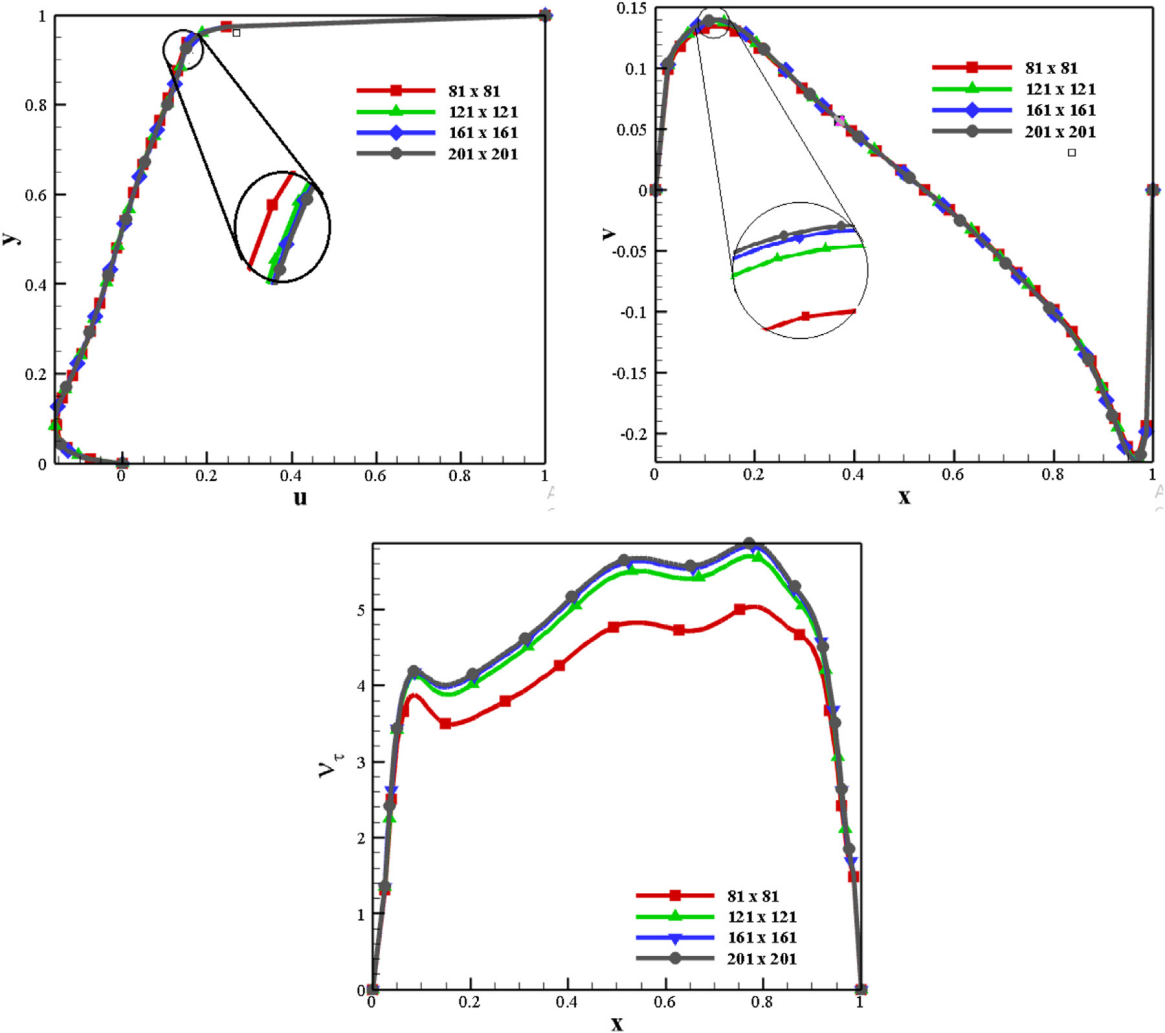
Grid Convergence Study.

As shown in Fig. 3, the grid convergence test of the current code is computed for various grid sizes 81 × 81, 121 × 121, 161 × 161, and 201 × 201 at *K* = 1.0. To ensure accurate grid sizes, the number of grids along the x and y-axes are equal. Contrasting the mid-plane velocities and turbulent viscosity explicitly proves that the 161 × 161 grid size can be selected for accuracy and computational time. Therefore, a 161 × 161 grid size has been used to further carry out the current numerical study. All results in the current investigation attained a maximum residual of 10^−8^.

#### Code validation study

**Fig. 4:**
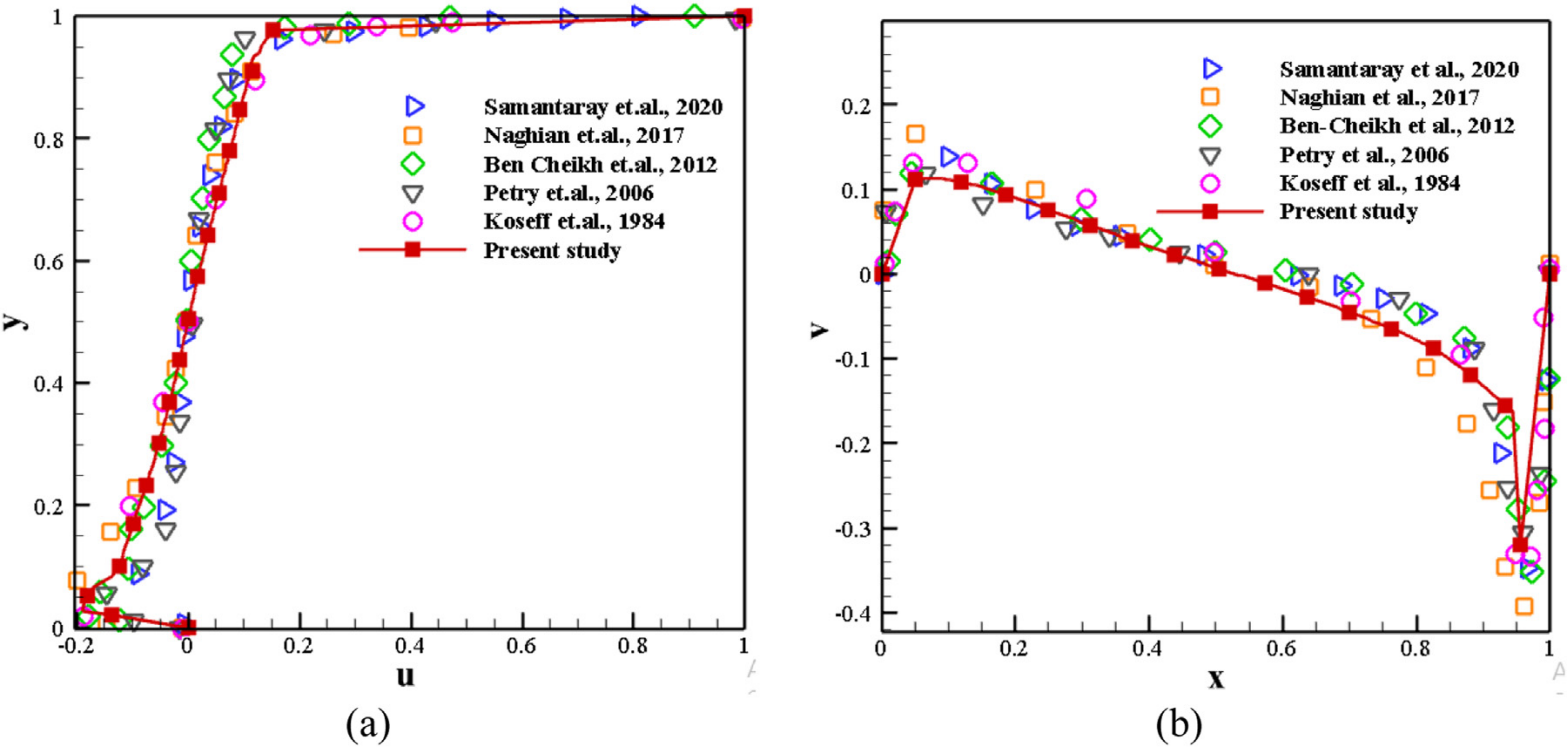
Centreline velocity profiles with uniform top wall motion compared with various literature at *Re*=1 × 10^4^, *K* = 1.0.

The single lid-driven flow is simulated using the FVM code to validate the current numerical research. Fig. 4 shows the evaluation of mid-plane u-velocity and v-velocity profiles at *Re*=1 × 10^4^ and *K* = 1.0, presenting numerical results compared with earlier literature [31,[52], [53], [54], [55] for top wall only moving with a velocity of U_T_=1. The excellent agreement between the existing and current studies establishes the simulation's validity.

### Results and discussion

#### Streamline contours for speed ratios and Reynolds numbers at *K*=1.0

**Fig. 5:**
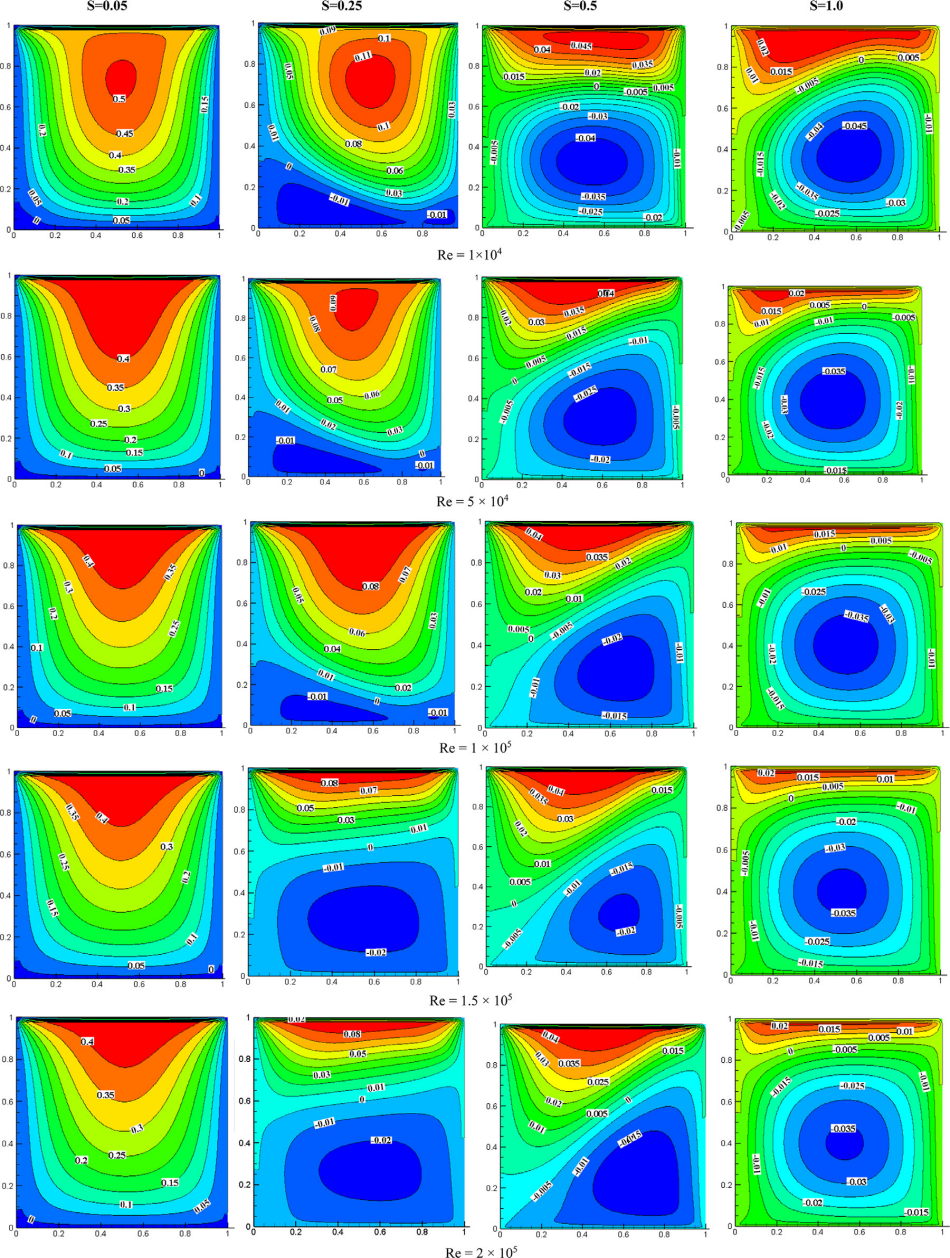
Streamline contours for different Speed ratios and Reynolds numbers at *K* = 1.0.

In Fig. 5, streamline contours are depicted for various Reynolds numbers and speed ratios at *K* = 1.0. At *S* = 0.05, the primary vortex occupies almost the entire cavity, while small secondary vortices are observed near the bottom wall due to the speed ratio's impact. As the speed ratio approaches 0.25, the secondary vortices grow in size. These secondary vortices merge and transform into the primary vortex when the speed ratio increases to 0.5, leading to the primary vortex decreasing in size and moving upward. When *S* = 1.0, the secondary vortex reaches its maximum size, while the primary vortex becomes taller and shrinks to its smallest size. Fig. 5 shows that variations in the Reynolds number alter the primary vortex's position. With an increase in Reynolds number, the primary vortex's center moves upward, and the secondary vortex's size slightly increases. By elevating the Reynolds number, the primary vortex's center shifts closer to the top wall of the cavity for all speed ratio.

#### Effect of Reynolds number on turbulent quantities at *S*=0.05, *K*=0.5

**Fig. 6:**
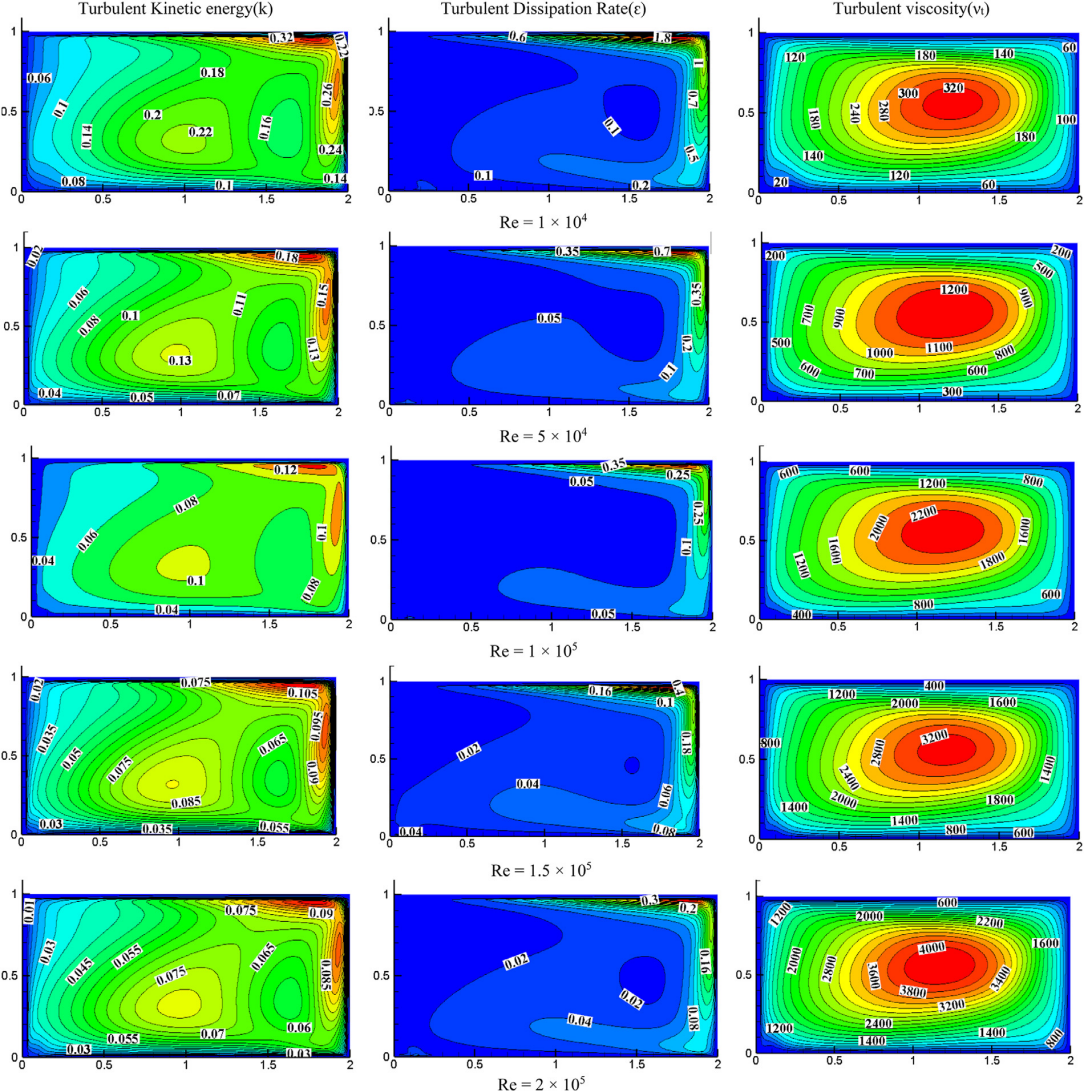
Impact of Reynolds number on turbulent quantities at *S* = 0.05, *K* = 0.5.

Fig. 6 depicts the Reynolds numbers effect on turbulent quantities for *K* = 0.5 and *S* = 0.05. The impact of turbulent kinetic energy (TKE) decreases as the *Re* increases. Because of the maximum pressure, the peak value is obtained close to the top right corner. Similarly, TKE is the least close to the cavity's left bottom. The dissipation rate is found to decrease with increasing Reynolds numbers. The turbulent dissipation rate is high at the cavity's top right because of higher TKE. The turbulent viscosity rises as the Reynolds number increases, in contrast to turbulent kinetic energy and dissipation. It indicates that as the flow became more turbulent. The intensity of non-dimensional turbulent viscosity varies from 320 to 4000 by increasing the *Re*.

#### Effect of Reynolds number on turbulent quantities for *S*=0.05, *K*=1.0

**Fig. 7:**
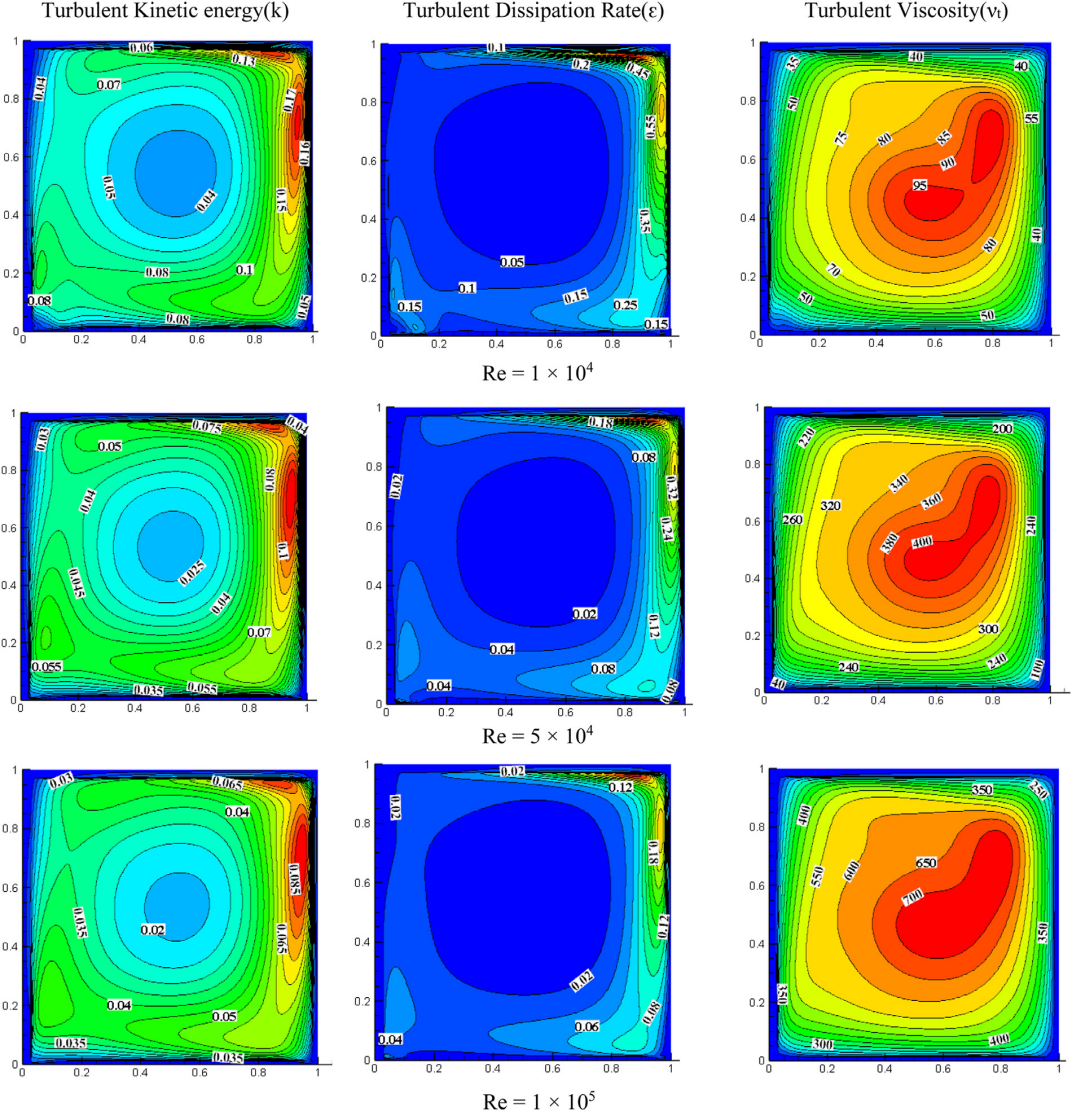

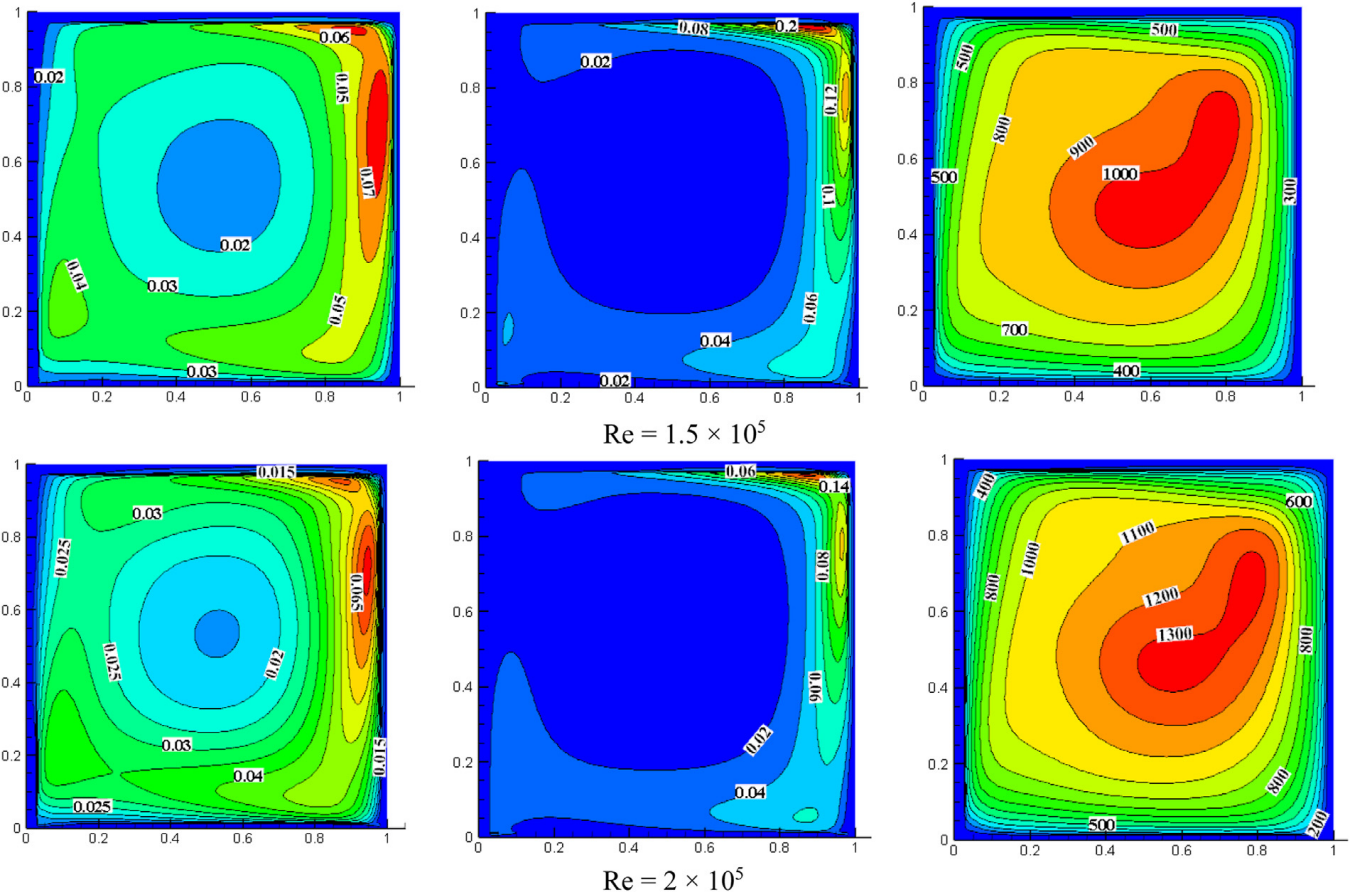
Impact of Reynolds number on turbulent quantities at *K* = 1.0, *S* = 0.05

Fig. 7 illustrates the impact of Reynolds numbers on turbulent quantities for *K* = 1.0 and *S* = 0.05. Increasing the Reynolds number decreases TKE, with the highest value observed near the top wall, while the lower part of the wall has a lower TKE. Higher TKE is observed on the cavity's right side due to the wall's X-direction movement. As the *Re* increases, the dissipation rate decreases, and both the dissipation rate and TKE peak at the top right of the cavity. In contrast, turbulent quantities rise with increasing Reynolds number, indicating a more turbulent flow.

#### Effect of Reynolds number on turbulent quantities for *S*=0.05, *K*=2.0

**Fig. 8:**
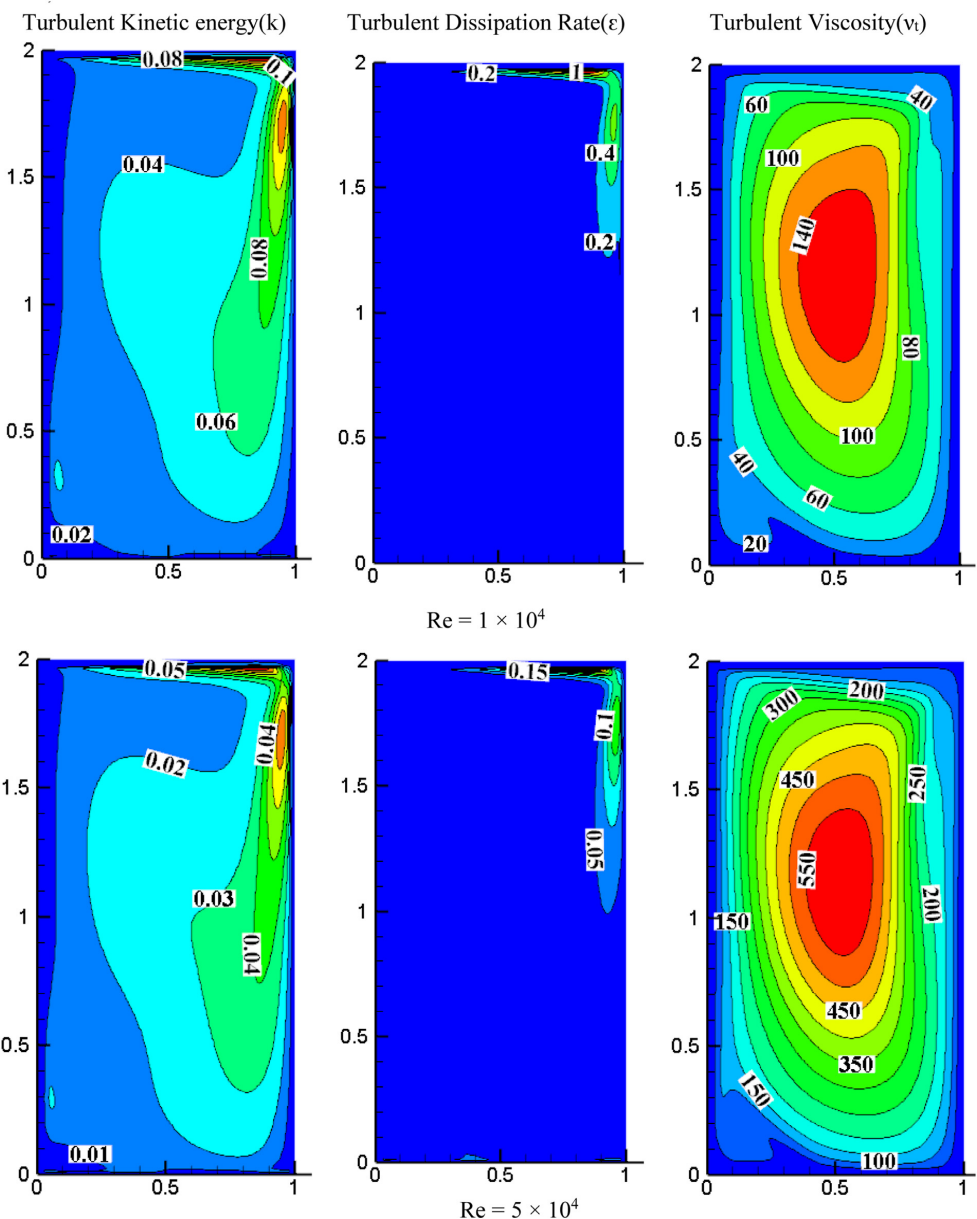

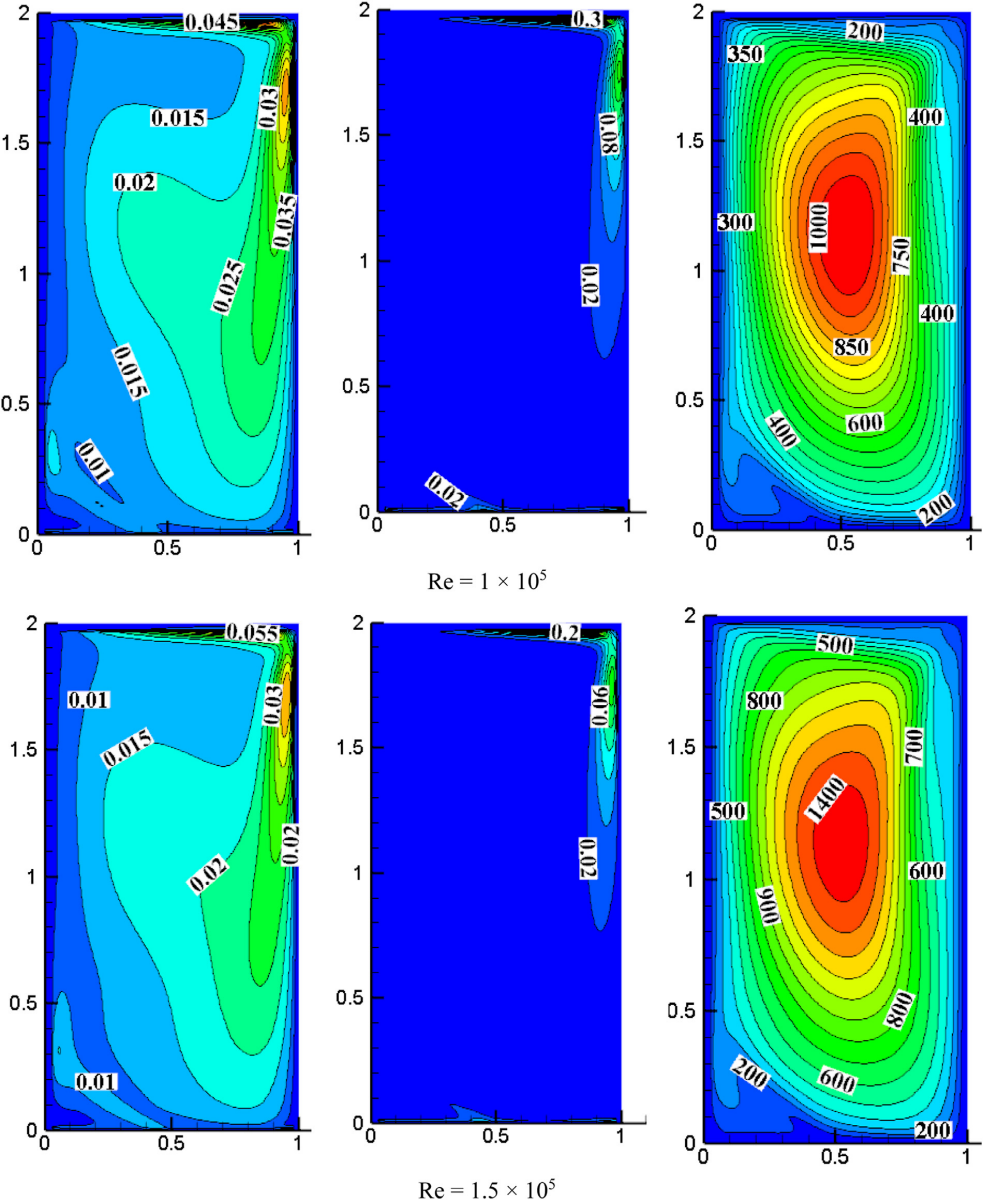

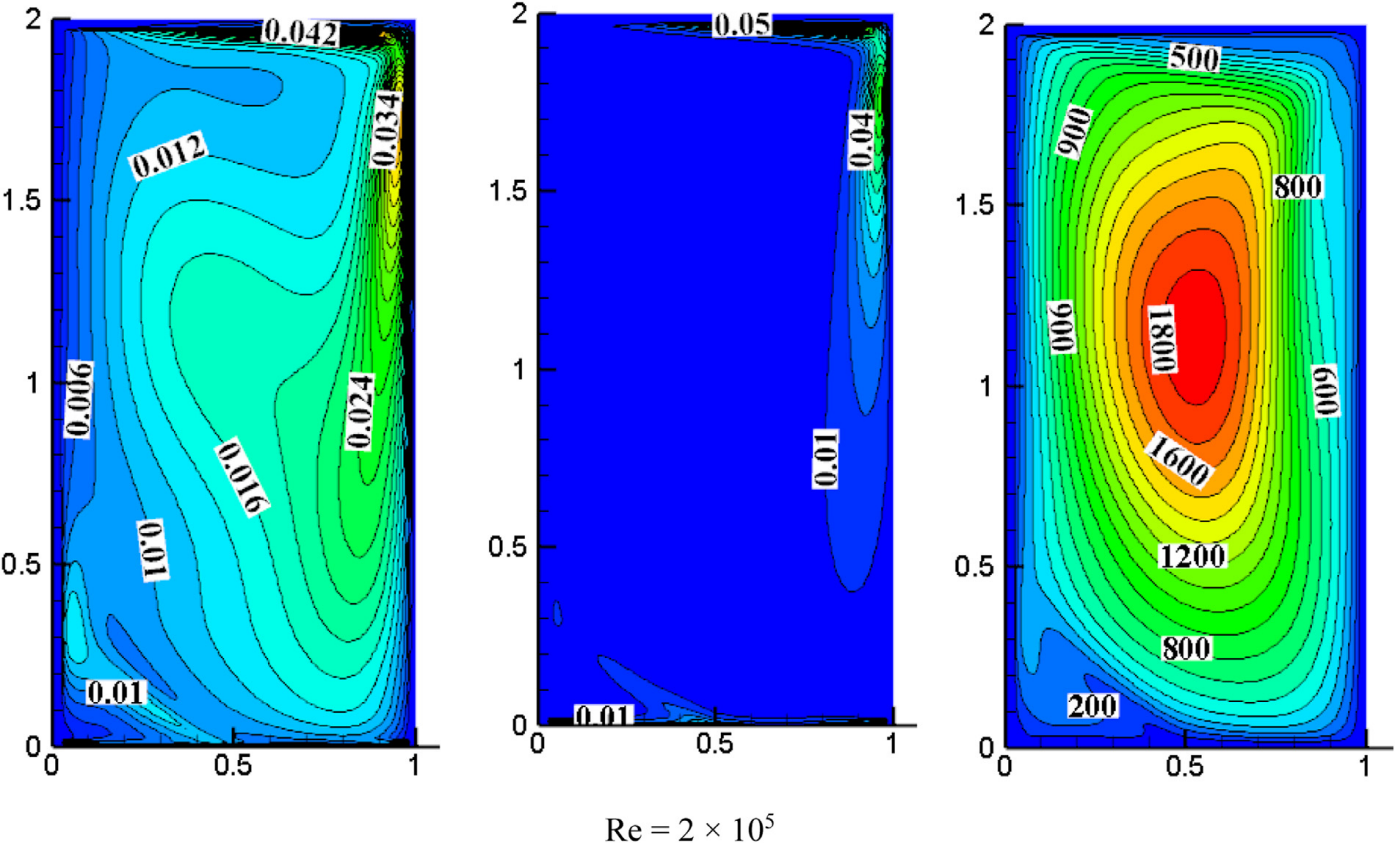
Impact of *Re* on turbulent quantities at *K* = 2.0, *S* = 0.05.

Fig. 8 shows the impact of the Reynolds number on turbulent quantities for *K* = 2.0, *S* = 0.8. For *Re*=1 × 10^4^, the intensity of TKE is more on the cavity's top right. Increasing *Re* increases the distribution of TKE, occupying the entire cavity. The turbulent dissipation rate's intensity is also higher in the top right of the cavity because the wall moves towards the right side and the higher velocity of the top wall (*S* = U_T_/U_B_). For *Re*=1 × 10^4^, the dissipation rate appeared only on the top right side, and the rest of the cavity is negligible while increasing *Re* magnitude is reduced, and the flow drifted more as compared with low *Re*. The turbulent dissipation rate is observed at the cavity's bottom when *Re* increases gradually. As the *Re* increases, the intensity of turbulent viscosity also increases. The maximum non-dimensional value of turbulent viscosity is increased from 140 to 1800 for a range of *Re* from 1 × 10^4^ to 2 × 10^5^.

For *S* = 0.05 and *Re*=2 × 10^5^, the impact of aspect ratio on turbulent quantities is presented in Figs. 6, 7, and 8. Increasing the aspect ratio results in a decrease in the concentration of TKE. The maximum TKE for *K* = 0.5 is located around the top right of the cavity. However, for *K* = 1.0, the intensity of TKE is reduced to 0.065 and shifted toward the right. However, the intensity is further diminished and dispersed throughout the cavity when the aspect ratio reaches 2.0. The rate of dissipation reduces with changes in aspect ratio. The value of TKE and dissipation rate are low in the bottom wall for *K* = 0.5 and *K* = 1.0, but for *K* = 2.0, the dissipation rate is also observed significantly near the center of the bottom wall. When *K* = 0.5, the middle region has the highest turbulent viscosity, 4000. The value dropped to 1300 and shifted slightly to the top right as the aspect ratio changed to 1.0. When *K* = 2.0, the value rises once more until it reaches the highest value of 1800, which is towards the cavity's center and appears slightly pushed downward.

#### Impact of mid-plane velocities for *Re* and S at *K*=1.0

**Fig. 9:**
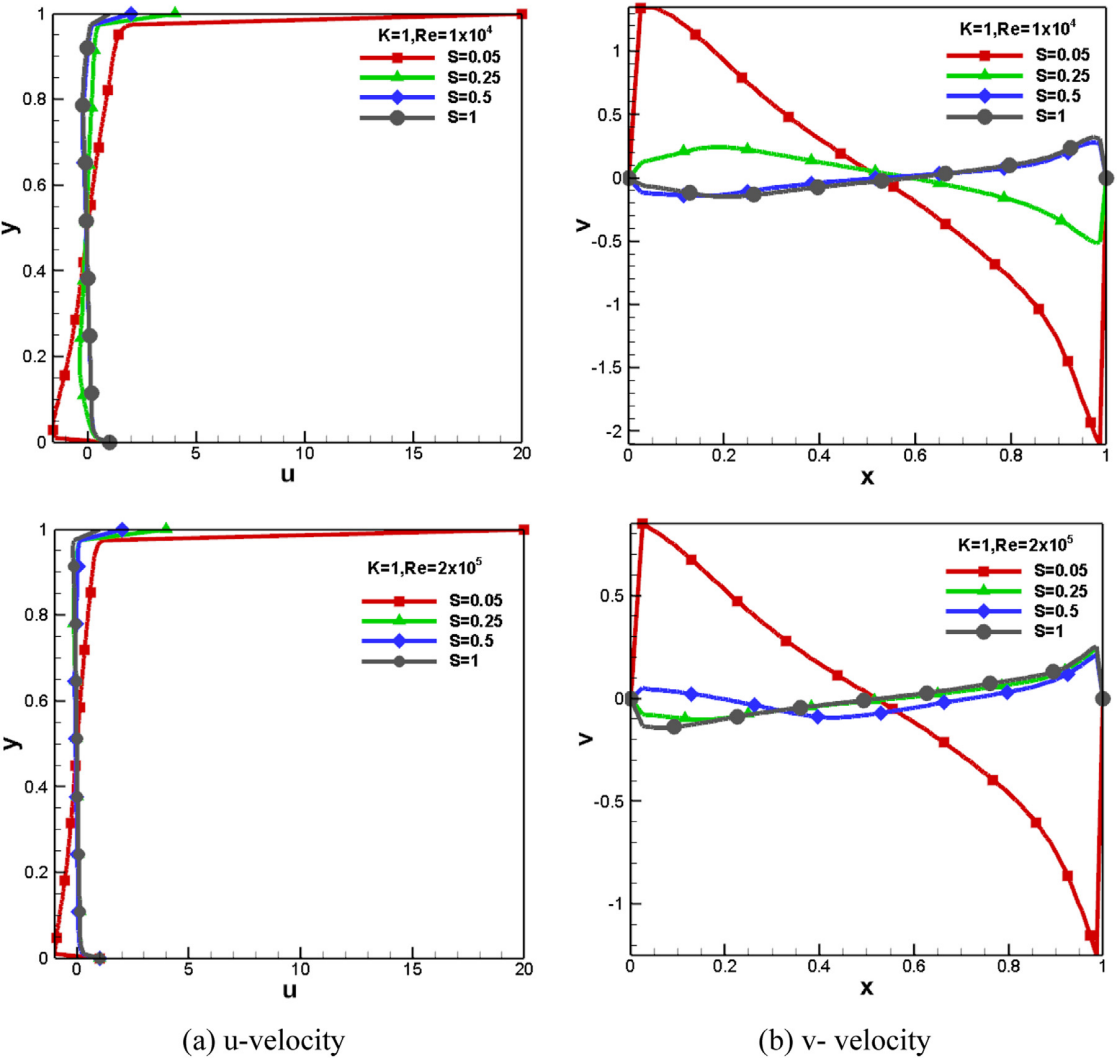
Impact of mid-plane velocities for *Re* and S at *K* = 1.0.

Fig. 9 shows the mid-plane u and v velocities for different speed ratios and *Re* at aspect ratio 1.0. At a low-speed ratio, the top wall moves at a non-dimensional u velocity of 20.0 (equal to *S* = 0.05). It is observed that horizontal velocity decreases from the top wall toward the cavity center. Only changing the speed ratio near the moving walls affects the fluid motion.

#### Impact of Reynolds stress for S and *Re* at *K*=0.5

**Fig. 10:**
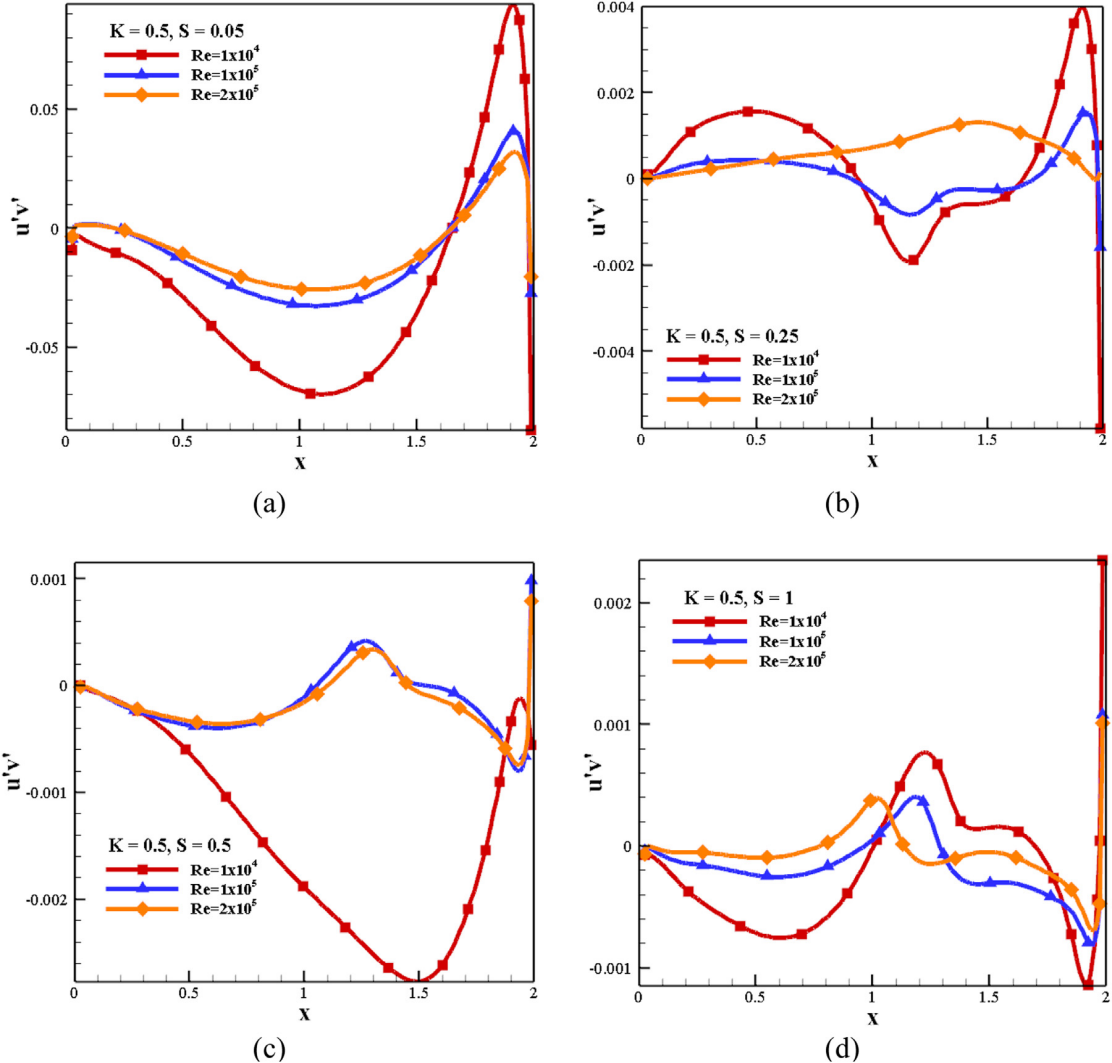
Impact of Reynolds stress (u'v') for S and *Re* with *K* = 0.5.

Fig. 10 shows the influence of the Reynolds stress for S and *Re* with *K* = 0.5. *Re*=1 × 10^4^ yields the Reynolds stress component's maximum fluctuation for all speed ratios. The intensity of Reynolds stress is decreased with an increase in speed ratio, and it is more on the right side due to the wall moving toward the right. It is also observed that the fluctuation component of Reynolds stress is decreased with an increase in *Re*.

#### Impact of TKE for S, K, and *Re*

**Fig. 11:**
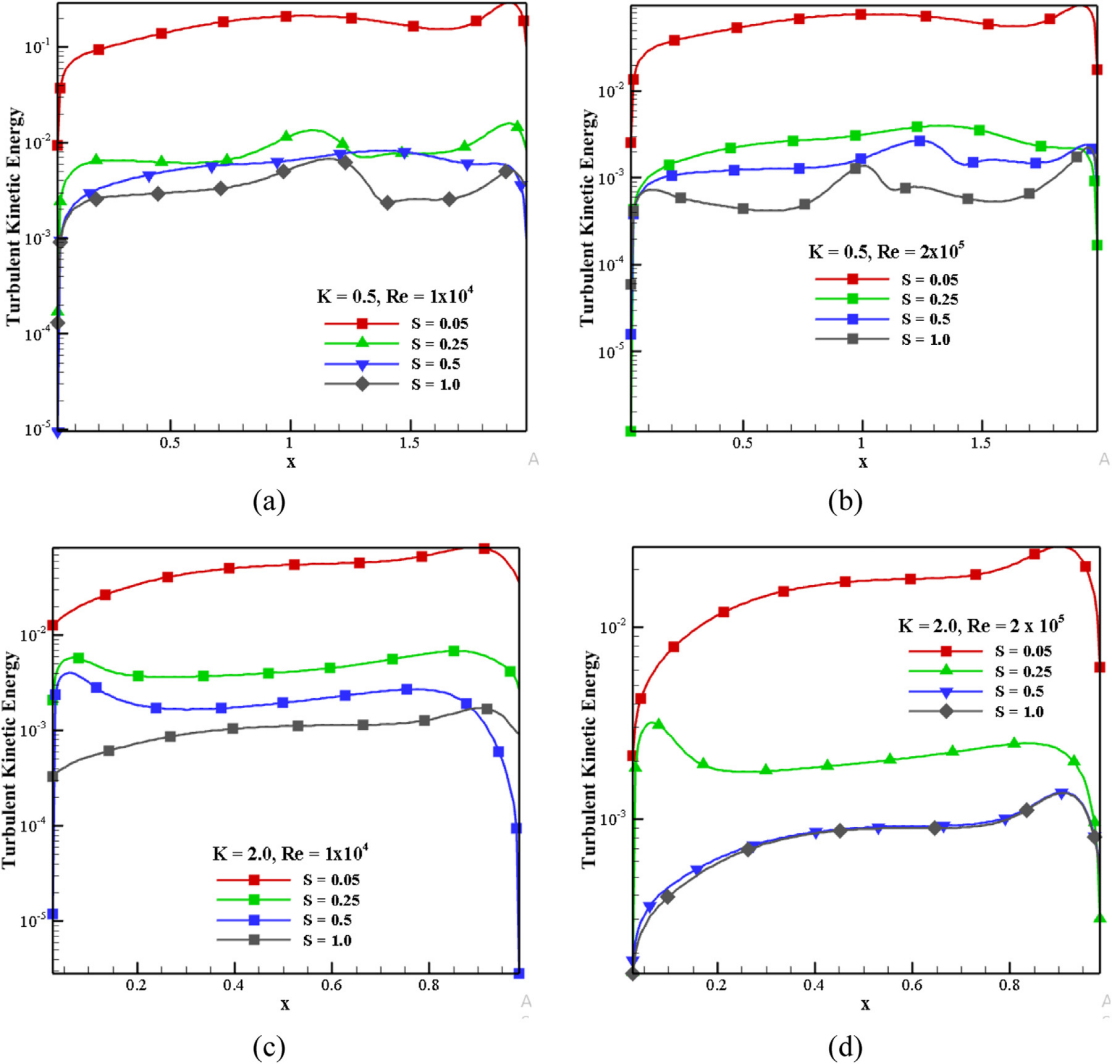
Impact of TKE in horizontal mid-plane for S, *Re*, K.

Fig. 11 shows the effect of TKE at the horizontal mid-plane for different S, *Re*, and K. It is shown in the log scale to quickly identify the speed ratio effects. For *K* = 0.5 and *Re*=1 × 10^4^, while varying the speed ratio from 0.05 to 1.0, the intensity of turbulent kinetic energy is gradually reduced, as shown in Fig. 11(a). TKE intensity is less on the left side and more on the right side because the wall moves towards the right. When *S* = 0.05, TKE intensity is higher for all aspect ratios. From Figs. 11(a) and (b) clearly show that the TKE decreased by 89.16% between *Re* =1 × 10^4^ and *Re*=2 × 10^5^ for *S* = 0.05. This observation applies to all speed ratios; when *Re* increases, the TKE decreases significantly. Similarly, TKE is reduced by around 73.52% with *K* = 0.5 and 2.0 for *Re*=1 × 10^4^and *S* = 1.0 (as shown in Figs. 11(a) and (c)). Figs. 11(b) and 11(d) compare the maximum TKE, which is decreased by around 72.96% for *K* = 0.5 and 2.0 for *Re*=2 × 10^4^ and *S* = 0.05.

#### Impact of turbulent dissipation rate for S, K, and *Re*

**Fig. 12:**
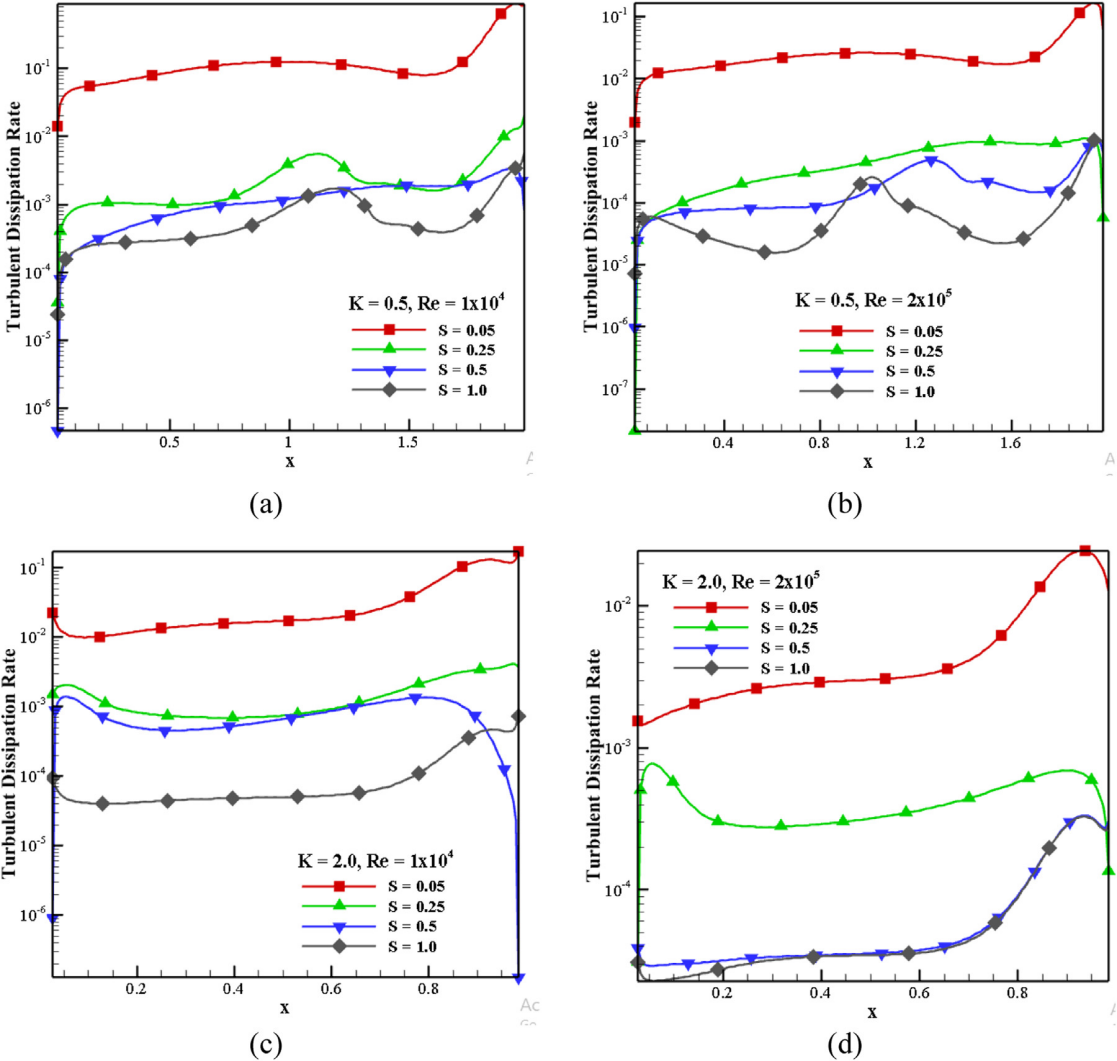
Effect of dissipation rate in horizontal mid-plane for different S, *Re*, K.

Fig. 12 shows the impact of turbulent dissipation rate in horizontal mid-plane for different S, *Re*, and K at +*X* direction. For *K* = 0.5 and *Re*=1 × 10^4^, the dissipation rate of turbulent flow decreases with an increase in S, as shown in Fig. 12(a). The dissipation rate is more in the direction of the moving wall and less on the left side of the cavity. From Fig. 12(a) and (b), the dissipation rate decreased by 42.28% for *Re*=1 × 10^4^ and *Re*=2 × 10^5^ at *S* = 0.05. With the increase in *Re*, the energy dissipation rate almost decreases with S. For *Re*=1 × 10^4^ and *S* = 1.0, the dissipation rate decreased by 87.01% with *K* = 0.5 and 2.0 (Figs. 12(a) and 12(c)). At *S* = 0.05 and *Re* = 2 × 10^5^, the dissipation rate near the right wall is decreased by around 85.37% for *K* = 0.5 and 2.0 (Figs. 12(b) and 12(d)).

#### Effect of turbulent viscosity for different S, K, and *Re*

**Fig. 13:**
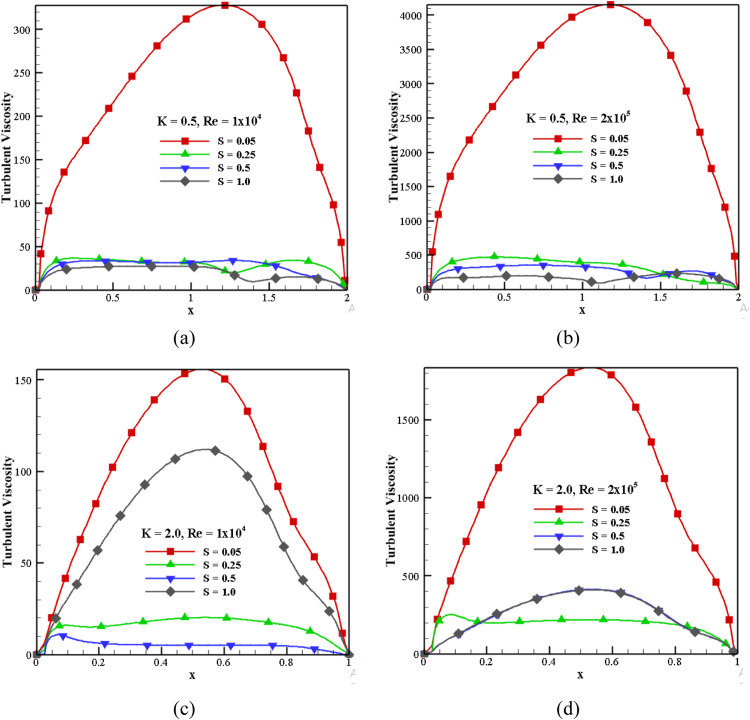
Effect of turbulent viscosity (ν_t_) in horizontal mid-plane for different S, *Re*, K.

Fig. 13 represents the turbulent viscosity distribution for the various speed ratios, aspect ratios, and *Re*. With an increased speed ratio, turbulent viscosity intensity is greatly reduced because the top wall velocity is reduced according to the speed ratio for *K* = 0.5 and *Re* = 1 × 10^4^. It is also observed that the turbulent viscosity increased 92.10% for *Re*=1 × 10^4^ to *Re*=2 × 10^5^ with *S* = 0.05, as shown in Figs. 13(a) and (b) follow the same pattern for all speed ratios. Hence, the distribution occurs in a recirculation zone inside the cavity. When *K* = 2.0 and *Re*=1 × 10^4^, the effect of viscosity is more for a low-speed ratio, which is reduced by increasing the speed ratio (shown in Fig. 13(c)). From that, the speed ratio and *Re* affect the distribution of turbulent viscosity. For *Re*=1 × 10^4^ and *S* = 1.0, the turbulent viscosity is decreased by 56.44% with *K* = 0.5 and 2.0 (Figs. 13(a) and 13(c)). The Reynolds number increases the peak value of turbulent viscosity by 150 to 1800 for *Re*=1 × 10^4^ and *Re*=2 × 10^5^ at *K* = 2.0 and *S* = 0.05, shown in Fig. 13(c) and (d). At *S* = 1.0 and *Re* = 2 × 10^5^, the turbulent viscosity is decreased 75.89% for *K* = 0.5 and 2.0 (Figs. 13(c) and (d)).

#### Impact of average turbulent quantities for *Re*, S, and k

**Fig. 14:**
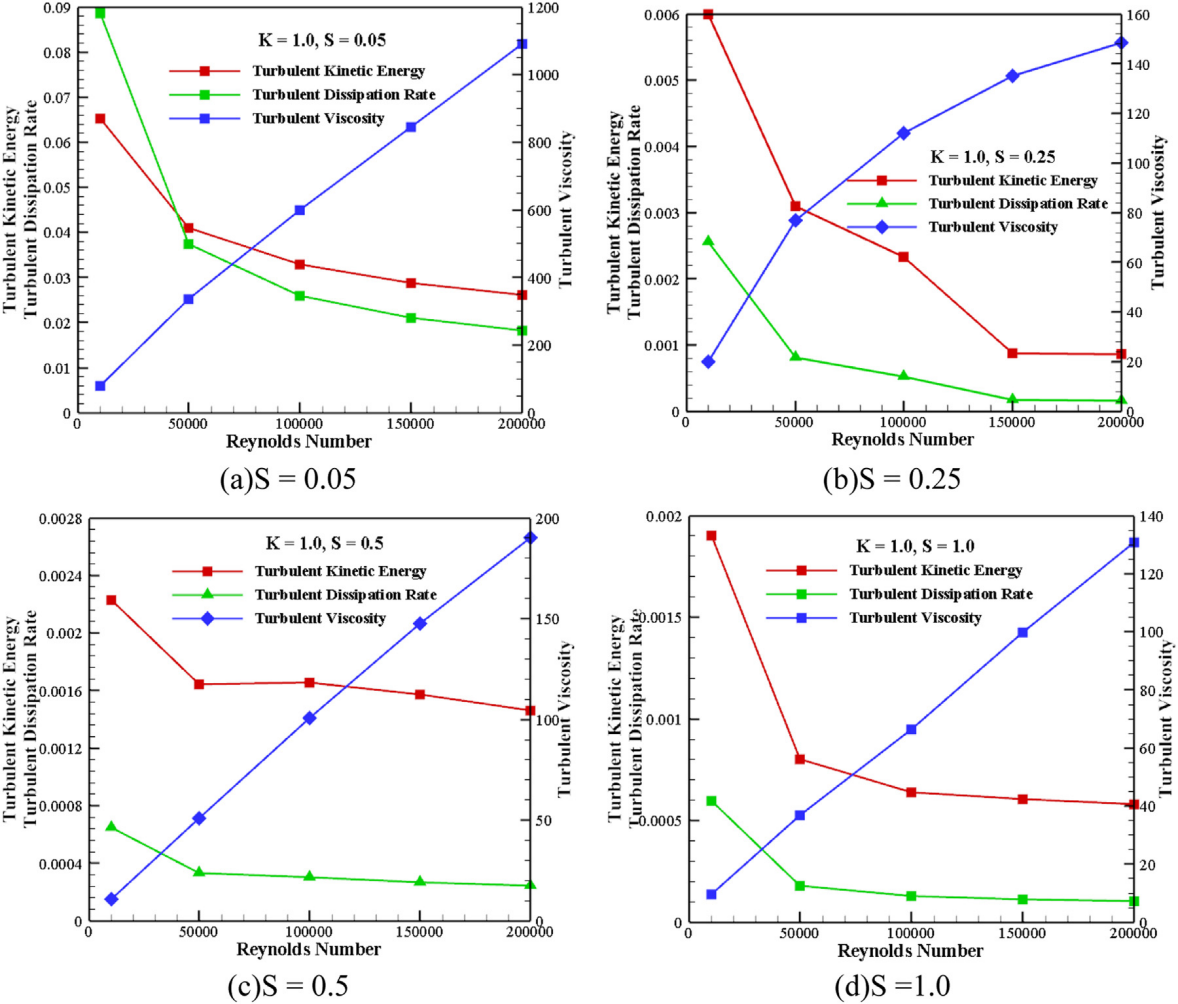
Impact of average turbulent quantities at *K* = 1.0.

Fig. 14 shows the impact of average turbulent quantities for all speed ratios and Reynolds numbers with *K* = 1.0. From Fig. 14(a), the average value of TKE and dissipation rate decreases by increasing the *Re*. It is also observed that the turbulent viscosity increases with an increase in *Re*. For the selected range of *Re*, the intensity of TKE and dissipation rate decreases by increasing the speed ratios, as depicted in Figs. 14(b-d). However, the turbulent viscosity intensity also decreases with an increased speed ratio.

### Conclusions

In this paper, a two-dimensional steady-state incompressible double-sided cavity fluid flow has been numerically studied by solving the governing equations over a wide range of operational parameters. The contours of streamline and turbulence quantities are analyzed. The key findings are summarized below.

• The analysis found that the speed ratio controls the lid motion's influence and strength of secondary corner eddies and determines the size of newly formed primary vortices and corner vortices.
• With an increase in flow parameters such as speed ratio, Reynolds number, and aspect ratio, there is a decrease in turbulent kinetic energy and dissipation and an increase in turbulent viscosity.
• For *S* = 0.05 and *K* = 0.5 with *Re* (1 × 10^4^–2 × 10^5^), turbulent kinetic energy and dissipation rate decreased by 89.16% and 42.28%, and turbulent viscosity increased by 92.10%.
• For *S* = 1.0 and *Re*=1 × 10^4^ with K (0.5–2.0) turbulent kinetic energy, the dissipation rate decreases by 73.52%, 87.01%, and turbulent viscosity increases by 75.44%.
• The above findings show that flow parameters increase when there is a decrease in average turbulent quantities.

### Ethics statements

None apply.

### CRediT author statement

Author 1 developed a code by discretizing the governing equations and conducted the numerical test for specified parametric conditions. The findings from these tests are presented as consolidated results for the current numerical problem. Author 2 has also developed the code and mathematical modeling section. He has compared the theoretical justifications with the literature. Finally, both authors revised the manuscript, and the final form was prepared per the journal format.

## Declaration of competing interest

The authors state that they do not have any known financial conflicts of interest or personal relationships that could have been perceived to affect the findings presented in this paper.

## Data Availability

Data will be made available on request.
